# Neutrophil extracellular traps induce a hypercoagulable state in glioma

**DOI:** 10.1002/iid3.488

**Published:** 2021-07-19

**Authors:** Shihua Zhang, Mengfan Guo, Qianzi Liu, Jingfeng Liu, Yankun Cui

**Affiliations:** ^1^ Department of Neurosurgery of the First Affiliated Hospital Jiamusi University Jiamusi China; ^2^ Department of Pathology of the First Affiliated Hospital Jiamusi University Jiamusi China; ^3^ Department of Pharmacy of Jiamusi University Jiamusi China; ^4^ Department of Outpatient of the First Affiliated Hospital Jiamusi University Jiamusi China

**Keywords:** endothelial cells, glioma, neutrophil extracellular traps, platelet, venous thromboembolism

## Abstract

**Background:**

Venous thromboembolism (VTE) is one of the leading complications in glioma patients. Neutrophil extracellular traps (NETs) have been reported to play a critical role in the physiopathology of cancer. We aimed to investigate the presence and potential role of NETs in the hypercoagulable state in glioma patients. Moreover, we evaluated the interaction between NETs and endothelial cells (ECs) in glioma patients.

**Methods:**

The plasma levels of NETs were detected by enzyme‐linked immunosorbent assay. The NET procoagulant activity was performed based on fibrin formation assays. The NET generation and NET‐treated ECs in vitro were observed by confocal microscopy. Activated platelets (PLTs) and PLT‐neutrophil aggregates were detected by flow cytometry.

**Results:**

Plasma NET markers were significantly higher in stage III/IV glioma patients than in stage I/II glioma patients and healthy subjects. PLTs from glioma patients tended to induce NET formation than those from healthy subjects. NETs contributed to the hypercoagulable state in glioma patients. After ECs were incubated with NETs isolated from stage III/IV glioma patients, they lost their intercellular connections and were converted into procoagulant phenotypes. Combining DNase I and activated protein C markedly decreased endothelial dysfunction.

**Conclusions:**

Our results showed the interaction between NETs and hypercoagulability in glioma patients. Targeting NETs may be a potential therapeutic and prevention direction for thrombotic complications in glioma patients.

## Highlights

• The plasma levels of NETs are increased in samples from high‐grade glioma.
• PLT induce the generation of NETs in glioma patients.
• NETs contribute to procoagulant in glioma patients and platelet activation and convert endothelial cells (ECs) to thrombogenicity.

## Graphical abstract

The plasma levels of neutrophil extracellular traps (NETs) are increased in samples from high‐grade glioma. PLT induces the generation of NETs in glioma patients. NETs contribute to procoagulant in glioma patients and platelet activation and convert endothelial cells (ECs) to thrombogenicity.

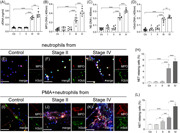

## 1 INTRODUCTION

Venous thromboembolism (VTE) is a frequent vascular complication in patients with cancer.^1^, ^2^, ^3^ VTE in cancer patients is associated with disease progression, poor prognosis, clinical deterioration, and in‐hospital mortality.^4^, ^5^ The rate of VTE varies with the type of cancer.^1^, ^2^, ^3^, ^4^, ^5^ The incidence of VTE in patients with high‐grade glioma (HGG) ranges from 20% to 30%.^6^, ^7^, ^8^ The risk of VTE in HGG patients is highest during the first few months after surgery and remains higher than other malignant tumors throughout the course of the disease.^9^, ^10^ However, the mechanism of VTE development in glioma patients remains unclear and complicated.

Neutrophils have been considered as key players in innate immunity.^11^ Numerous clinical and basic studies have proved the potential role of neutrophils in cancer pathophysiology.^12^, ^13^, ^14^ Neutrophil counts have previously been associated with the prognosis of glioma patients.^15^, ^16^, ^17^, ^18^, ^19^ Neutrophil extracellular traps (NETs) have been recently reported in glioma tissues and their expression was correlated with glioma grades.^20^ NETs are web‐like DNA‐containing structures released from activated neutrophils, which are comprised of decondensed chromatin and proteases such as myeloperoxidase (MPO) and neutrophil elastase (NE).^21^, ^22^, ^23^ However, the presence of NETs in the plasma from glioma patients and their potential association with cancer development is not well elucidated. Several studies have reported that leukocytosis is associated with cancer‐associated thrombosis and NETs may act as a bridge between cancer and thrombosis.^24^, ^25^, ^26^, ^27^, ^28^ Whether NETs participate in the procoagulant activity (PCA) in glioma patients has not been investigated. Endothelial injury has been recognized as a critical cause of thrombosis.^29^, ^30^ NETs have been reported to induce endothelial dysfunction in some diseases.^31^, ^32^, ^33^, ^34^ However, the interaction between NETs and endothelial injury in glioma patients has not been fully understood.

In this study, we hypothesized that high plasma levels of NETs were correlated with thrombogenicity in glioma patients. Hence, we detected NETs markers in the plasma from glioma patients and healthy subjects and investigate the interaction between NETs and platelets (PLTs). In addition, we evaluated the PCA of NETs from glioma patients and endothelial cells (ECs) incubated with NETs. Our results may offer potential therapeutic targets for thrombotic complications in glioma patients.

## 2 MATERIALS AND METHODS

### 2.1 Patients

Thirty glioma patients who were admitted to the First Affiliated Hospital of Jiamusi University between October 2015 and June 2020 were enrolled. The diagnosis of all patients was confirmed by pathological examinations after surgery. Blood samples of all patients were obtained before surgery. Exclusion criteria were age <18 years, pregnancy, cardiovascular disease, liver or renal dysfunction, other thromboembolic complications, PLTs and/or blood coagulation disorders and administration of anticoagulant and/or antiplatelet treatment. The main clinical characteristics of the patients and healthy controls are shown in Table 1. This study was approved by the research ethics committee of Jiamusi University and informed consent was obtained from all participants.

**Table 1:** Characteristics of the study population

Characteristics	Control (*n* = 13)	Stage I (*n* = 7)	Stage II (*n* = 8)	Stage III (*n* = 8)	Stage IV (*n* = 7)
Age (years)	35.07 ± 0.5	35.57 ± 4.0	43.63 ± 0.5	40.88 ± 10.5	53.43 ± 9.5
Male (*n*, %)	38.4%	42.8%	28.5%	50%	71.4%
WBC (×10^9^)	4.11 ± 1.02	4.05 ± 2.12	4.13 ± 0.72	4.15 ± 0.56	4.02 ± 0.72
Neutrophils (%)	65.62 ± 9.43	66.34 ± 7.32	64.45 ± 5.32	65.57 ± 8.27	68.87 ± 5.23*
Monocytes (%)	2.38 ± 1.06	2.44 ± 1.26	2.37 ± 1.02	2.40 ± 1.12	2.38 ± 1.42
Lymphocytes (%)	15.38 ± 8.87	15.44 ± 7.54	16.02 ± 6.43	15.75 ± 5.35	15.66 ± 4.54
Esoinophils (%)	0.30 ± 0.47	0.29 ± 0.45	0.30 ± 0.50	0.29 ± 0.54	0.30 ± 0.44
Basophils (%)	0	0	0	0	0
Erythrocytes (×10^12^/L)	3.92 ± 0.51	3.94 ± 0.44	3.90 ± 0.45	3.94 ± 0.50	3.91 ± 0.66
Hg (g/L)	123 ± 18.5	122 ± 13.4	122 ± 14.1	125 ± 11.2	124 ± 14.8
PLT (×10^9^)	234 ± 45.6	238 ± 40.5	244 ± 32.3	241 ± 28.4	239 ± 42.2
PT (s)	12.3 ± 0.9	12.0 ± 0.8	12.2 ± 0.9	11.9 ± 0.8	12.2 ± 1.0
APTT (s)	32.4 ± 2.3	32.5 ± 1.9	33.2 ± 2.0	33.1 ± 2.0	32.6 ± 1.3
d‐dimer (mg/L)	357.79 ± 3.7	334.45 ± 5.9	366.74 ± 8.7	348.64 ± 9.9	359.44 ± 7.4
Fibrinogen (mg/L)	2.74 ± 0.77	2.69 ± 0.85	2.70 ± 0.65	2.58 ± 0.87	2.77 ± 0.75

*Note*: The main clinical and laboratory features of 13 healthy subjects and 30 patients diagnosed with glioma. Data are presented as numbers (percentages) or the mean ± SD.

Abbreviations: Hb, hemoglobin; PLTs, platelets; WBC, white blood cells.

* *p* < .05 versus healthy control.

### 2.2 Human samples

Neutrophils were isolated from fresh citrated whole venous blood samples using the human whole blood neutrophil isolation kit (TBD; Tianjin) according to the product instructions. For PLTs isolation, citrated blood was centrifuged at 150 g for 17 min and PLT‐rich plasma (PRP) was obtained. Seventy‐five percent of the top layer was removed and diluted with PLT wash buffer (TBD; Tianjin), followed by centrifugation at 460*g* for 17 min at room temperature. Isolated PLTs were resuspended in pre‐warmed Tyrode's buffer (Solarbio). For PLT poor plasma, blood samples were centrifuged at 1000*g* for 10 min, followed by centrifugation of the supernatant at 15,000*g* for 20 min as previously described.^31^

### 2.3 NETs isolation and quantification

NETs were isolated from healthy subjects and glioma patients as previously described.^25^, ^31^ The quantification of cell‐free DNA (cf‐DNA) was performed by the Quant‐iT PicoGreen dsDNA assay kit (Invitrogen).^31^, ^35^ For quantification of NET complexes, MPO enzyme‐linked immunosorbent assay (ELISA) kit (Jianglaibio), NE ELISA kit (Jainglaibio), and citH3 ELISA kit (Jianglaibio) were combined with Quant‐iT PicoGreen dsDNA assay kit (Invitrogen) respectively to detect MPO‐DNA, NE‐DNA, and citH3‐DNA as previously described.^31^, ^35^ For detecting TAT‐complex, 100 μl of NETs (0.5 μg DNA/ml) were incubated with 50 μl of pooled platelet‐poor human plasma, 50 μl of prewarmed 25 mmol/L CaCl_2_ for 2 h and then measured by TAT‐complex ELISA kit (Jingkang).

### 2.4 EC stimulation assays

Human umbilical vein endothelial cells (HUVECs) were incubated with different concentrations of NETs for 2 h and then treated ECs and fibrin formation were detected as previously described.^36^ For inhibition assays, isolated NETs (0.5 μg/ml) were added to HUVECs in the presence of DNase I and activated protein C (APC, 100 nM) and incubated for 2 h. Fibrin formation was performed as previously described.^36^

### 2.5 Flow cytometry

To detect PLTs phosphatidylserine (PS) exposure, whole blood or PLTs isolated from healthy subjects and glioma patients were stained with fluorescein isothiocyanate (FITC)‐conjugated bovine lactadherin (Haematologic Technologies) and PerCP‐Cy5.5‐conjugated‐CD41 (Biolegend). To investigate PLTs‐neutrophil aggregates, isolated neutrophils from glioma patients and healthy donors were incubated with FITC‐conjugated‐CD41 (Biolegend) and APC‐conjugated‐CD66b (Biolegend) antibodies. The flow cytometry strategy is in Supporting Information Data S1.

### 2.6 Immunofluorescence (IF) imaging

For NET staining, neutrophils were fixed and stained with 4′,6‐diamidino‐2‐phenylindole (DAPI), anti‐Histone H3 citrulline (R2 + R8 + R17, ab5103, 1:1000) and anti‐MPO (ab25989, 1:1000), followed by a goat anti‐rabbit secondary antibody conjugated to Alexa Fluor 594 (Proteintech, 1:200) and a goat anti‐mouse secondary conjugated to Alexa Fluor 488 (Proteintech, 1:200). To visualize PLT binding with NETs, treated NETs were stained with anti‐CD66b (Affinity, 1:500) and anti‐CD41 (Novus, 1:500), followed by a goat anti‐rabbit secondary antibody conjugated to Alexa Fluor 594 (Proteintech, 1:200) and a goat anti‐mouse secondary antibody conjugated Alexa Fluor 488 (Proteintech, 1:200). ECs cultured on fibronectin‐coated slide flasks were stained with anti‐VE‐cadherin (ab33168, 1:500), anti‐ZO‐1 (Proteintech, 1:500), and DAPI. All the IF images were analyzed by confocal microscopy. Quantification of mean fluorescence intensity was performed by ImageJ.

### 2.7 Statistical analysis

Data are shown as the mean ± standard deviation (SD). Statistical analysis was performed using Student's *t* tests, paired *t* tests, analysis of variance (ANOVA). All analyses were performed with Prism 8.0 (GraphPad). A *p* < .05 was considered statistically significant.

## 3 RESULTS

### 3.1 Plasma levels of NET markers are higher in glioma patients

**Figure 1:**
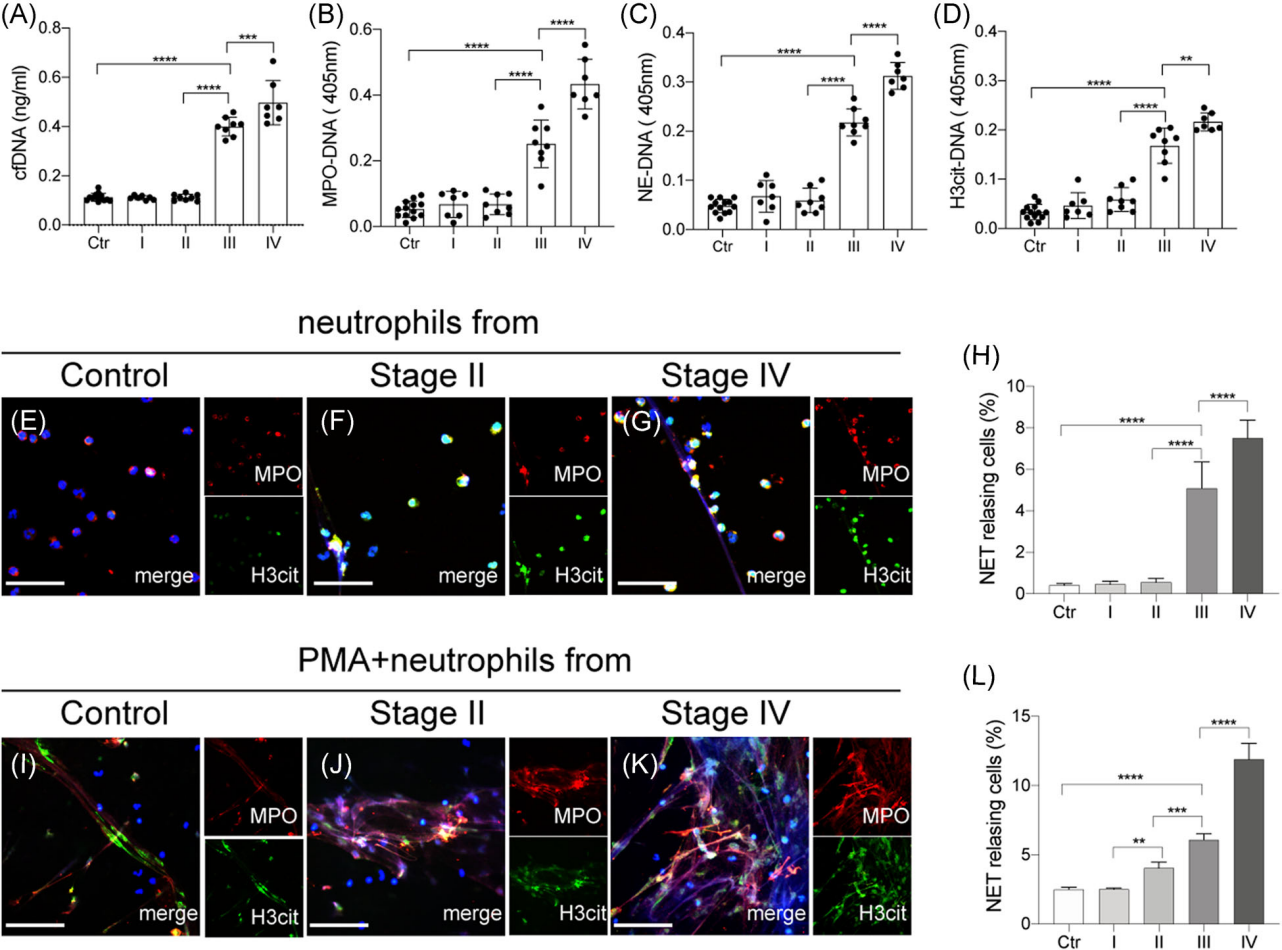
Neutrophil extracellular trap (NET) markers are increased in high‐grade glioma patients. NET markers, cf‐DNA (A), MPO‐DNA (B), NE‐DNA (C), and citH3‐DNA (D), were measured in plasma from healthy subjects and glioma patients. (E, F) Neutrophils from glioma patients and healthy subjects were incubated for 2 h in vitro, stained with MPO (green) and citH3 (red), and analyzed by confocal microscopy. (H) The rate of NETing neutrophils from each group. (I–K) Neutrophils from glioma patients and healthy subjects were incubated with PMA (25 nM) for 2 h in vitro, stained with MPO (green) and citH3 (red), and analyzed by confocal microscopy. (L) The rate of NETing neutrophils from each group. The scale bar in (E–G) and (I–K) are 40 μm. The results are expressed as the mean ± SD. *p < .05, **p < .01, ***p < .001 and ****p < .0001. MPO, myeloperoxidase; PMA, Phorbol‐12‐myristate‐13‐acetate

To investigate the presence of NETs in glioma patients, NET markers, cf‐DNA, MPO‐DNA, NE‐DNA, and citH3‐DNA, were detected in plasma from four stages of glioma patients (Figure 1A–D). Plasma levels of NET markers from stage III/IV patients were significantly higher than those from stage I/II patients and healthy subjects. Moreover, there were no significant differences in NET levels between stage I/II patients and healthy subjects. Neutrophils isolated from each glioma stage were stained with MPO and citH3 and analyzed by confocal microscopy (Figure 1E–H). Then control neutrophils isolated from each glioma stage were incubated with Phorbol‐12‐myristate‐13‐acetate (25 nM) and stained with MPO and citH3 and analyzed by confocal microscopy (Figure 1I–L). Our results showed similar trend indicating that neutrophils from stage III/IV glioma patients were more prone to release NETs than those from I/II glioma patients and healthy subjects.

### 3.2 PLTs initiate NET generation in glioma patients

**Figure 2:**
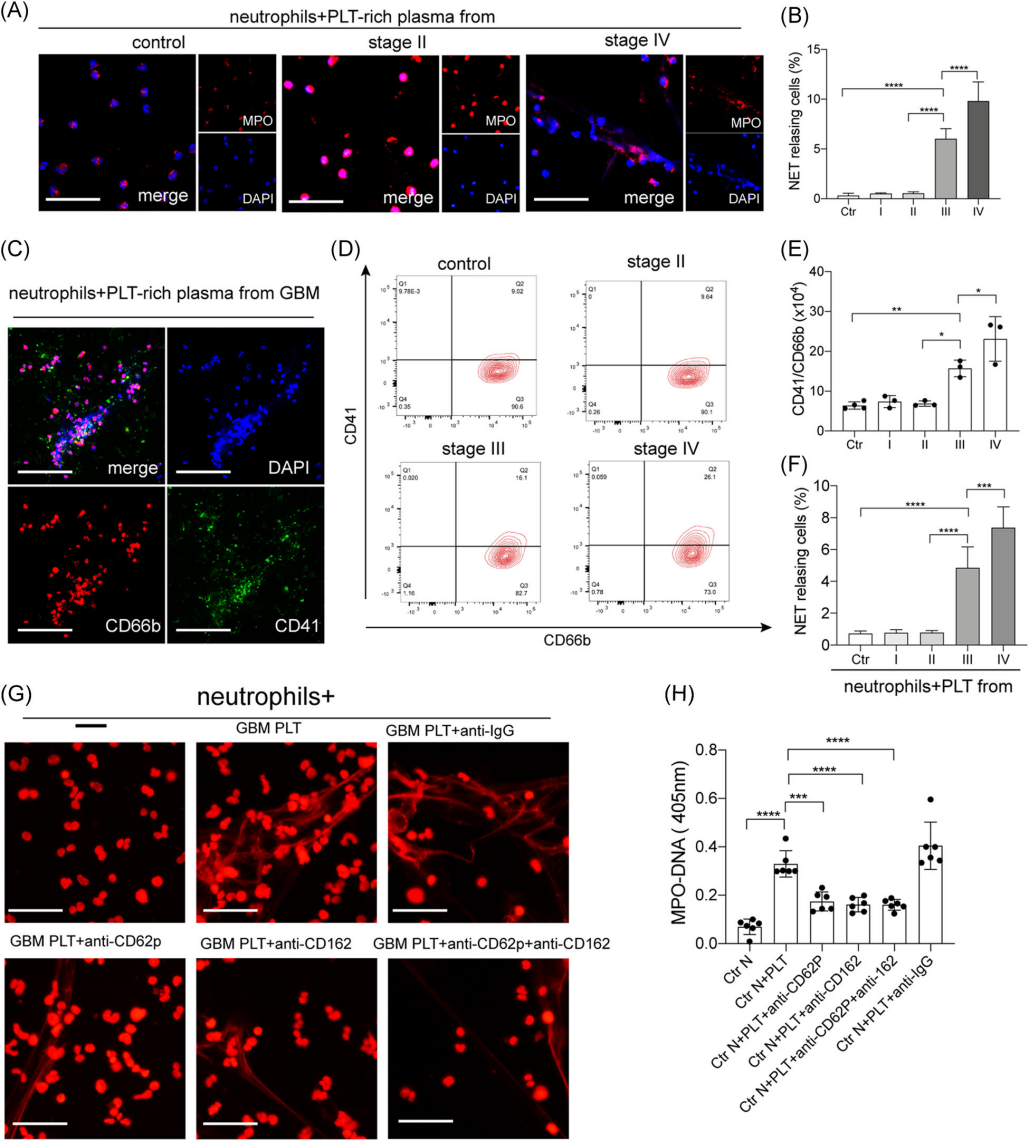
Platelet‐neutrophil interaction triggers NET generation in glioma patients. (A) Control neutrophils were incubated with PLT‐rich plasma from glioma patients and healthy subjects and stained with MPO (red) and DAPI (blue). (B) Plasma from stage III/IV glioma patients activated neutrophils to release higher proportions of NETs than those from stage I/II glioma patients and healthy subjects. (C) Immunofluorescence images also showed PLTs (CD41, green) decorated on DNA traps (DAPI, blue) when neutrophils (CD66b, red) were incubated with plasma from glioblastoma (GBM; stage IV glioma) patients. (D) PLT‐neutrophil aggregates, defined as CD41+/CD66b+ cells, were investigated in samples from each group. (E) The rate of PLT‐neutrophil aggregates was highest in samples from GBM patients. (F) Control neutrophils were incubated with PLTs from glioma patients and healthy subjects and analyzed by immunofluorescence microscopy. PLTs from stage III/IV glioma patients, especially GBM patients, were potent activators of NET formation than those from stage I/II glioma patients and healthy subjects. (G, H) Anti‐P‐selectin and PSGL‐1 antibodies were used to inhibit the interaction between neutrophils and PLTs and the NET (propidium iodide, red) formation was markedly decreased. The scale bar in (A) and (G) is 20 μm and (C) is 80 μm. The results are expressed as the mean ± SD. *p < .05, **p < .01, ***p < .001, and ****p < .0001. DAPI, 4′,6‐diamidino‐2‐phenylindole; MPO, myeloperoxidase; NET, neutrophil extracellular trap; PLT, platelet

To investigate the initiators of NET generation in glioma patients, neutrophils were treated with PRP from glioma patients. Confocal images showed that neutrophils treated with plasma from stage IV glioma patients generated a higher proportion of NETs than those from stage I/II glioma patients and healthy subjects (Figures 2A and 2B). IF images also showed PLTs decorated on DNA traps when neutrophils were incubated with plasma from glioblastoma (GBM; stage IV glioma) patients (Figure 2C). Therefore, we wondered whether PLTs participate in the generation of NETs in glioma patients. We investigated PLT‐neutrophil aggregates, defined as CD41+/CD66b+ cells, in samples from glioma patients. The proportion of PLT‐neutrophil aggregates was highest in samples from GBM patients (Figures 2D and 2E). Then control neutrophils were incubated with PLTs from glioma patients and healthy subjects and analyzed by IF microscopy (Figure 2F). PLTs from stage III/IV glioma patients, especially GBM patients, were more potent activators of NET formation than those from stage I/II glioma patients and healthy subjects. Then we used anti‐P‐selectin and PSGL‐1 antibodies to inhibit the interaction between neutrophils and PLTs (Figures 2G and 2H). The NET formation was markedly decreased when control neutrophils were pretreated with anti‐P‐selectin and PSGL‐1 antibodies, suggesting that PLTs are important initiators in NET generation in glioma patients.

### 3.3 NETs contribute to the PCA in glioma patients

**Figure 3:**
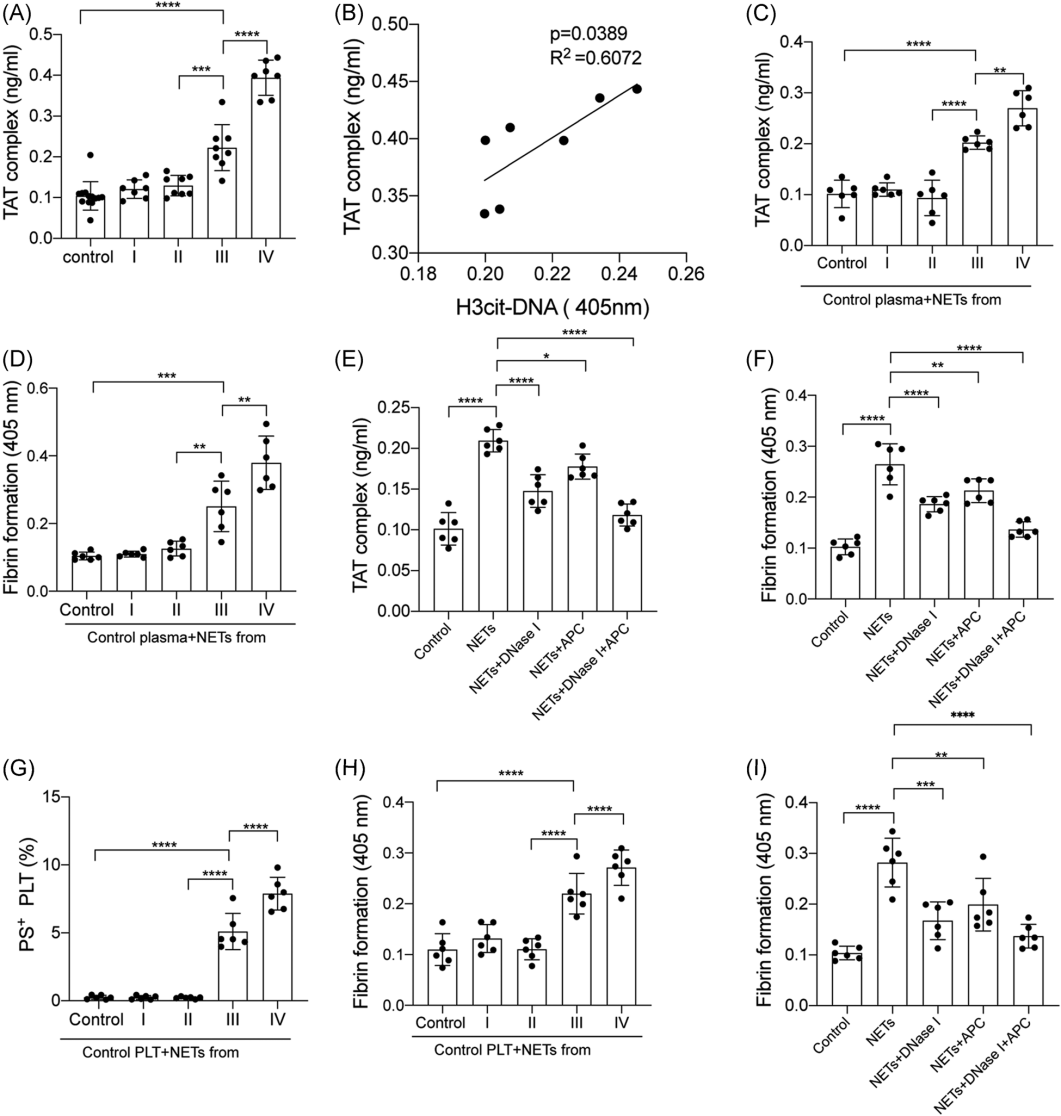
NETs participate in the procoagulant activity in glioma patients. (A) Thrombin‐antithrombin (TAT) complex in plasma from glioma patients and healthy subjects were measured by ELISA. (B) The TAT complex was correlated with NET markers citH3‐DNA in plasma from GBM patients. NETs isolated from neutrophils from GBM patients suggested higher levels of TAT‐complex (C) and fibrin formation (D) than those isolated from the other groups. In inhibition assays, isolated NETs from GBM patients were incubated with control plasma in the presence of DNase I and APC. The generation of TAT‐complex (E) and fibrin formation (F) were decreased by NETs inhibitors. Control PLTs were incubated with NETs from glioma patients and healthy subjects. (G) Flow cytometry showed a higher expression of phosphatidylserine (PS) in PLTs incubated with NETs from stage III/IV glioma patients than those from stage I/II glioma patients. (H) Fibrin formation was detected in PLTs incubated with NETs from each group. (I) In the presence of DNase I and activated protein C (APC), the fibrin formation of PLTs was elevated and close to that from healthy subjects. The results are expressed as the mean ± SD. *p < .05, **p < .01, ***p < .001, and ****p < .0001. ELISA, enzyme‐linked immunosorbent assay; GBM, glioblastoma; NET, neutrophil extracellular trap; PLT, platelet

Thrombin‐antithrombin (TAT) complex in plasma from glioma patients and healthy subjects were measured by ELISA. The levels of the TAT complex in plasma from stage III/IV glioma patients were significantly elevated than those from stage I/II glioma patients and healthy subjects (Figure 3A). Then we investigated the interaction between NETs and the coagulation state in glioma patients. The TAT complex was correlated with NET marker，citH3‐DNA in plasma from GBM patients (Figure 3B). Moreover, NETs isolated from neutrophils from GBM patients suggested higher levels of TAT‐complex and fibrin formation than those isolated from the other groups (Figures 3C and 3D). In inhibition assays, isolated NETs from GBM patients were incubated with control plasma, in the presence of DNase I and APC. Our results showed that NET inhibitors, DNase I and APC, significantly inhibited TAT‐complex and fibrin formation (Figures 3E and 3F). Flow cytometry showed the expression of PS was higher in PLTs from stage III/IV glioma patients than those from stage I/II glioma patients (Supporting Information Data S2A, B). Furthermore, control PLTs were incubated with NETs from glioma patients. PS expression was higher in PLTs incubated with NETs from stage III/IV glioma patients than those from stage I/II glioma patients (Figure 3G). We also observed a similar trend in detecting fibrin formation from these treated PLTs (Figure 3H). In addition, control PLTs were incubated with NETs from GBM patients and fibrin formation was markedly decreased in the presence of DNase I and APC (Figure 3I).

### 3.4 NETs convert ECs to a procoagulant phenotype

**Figure 4:**
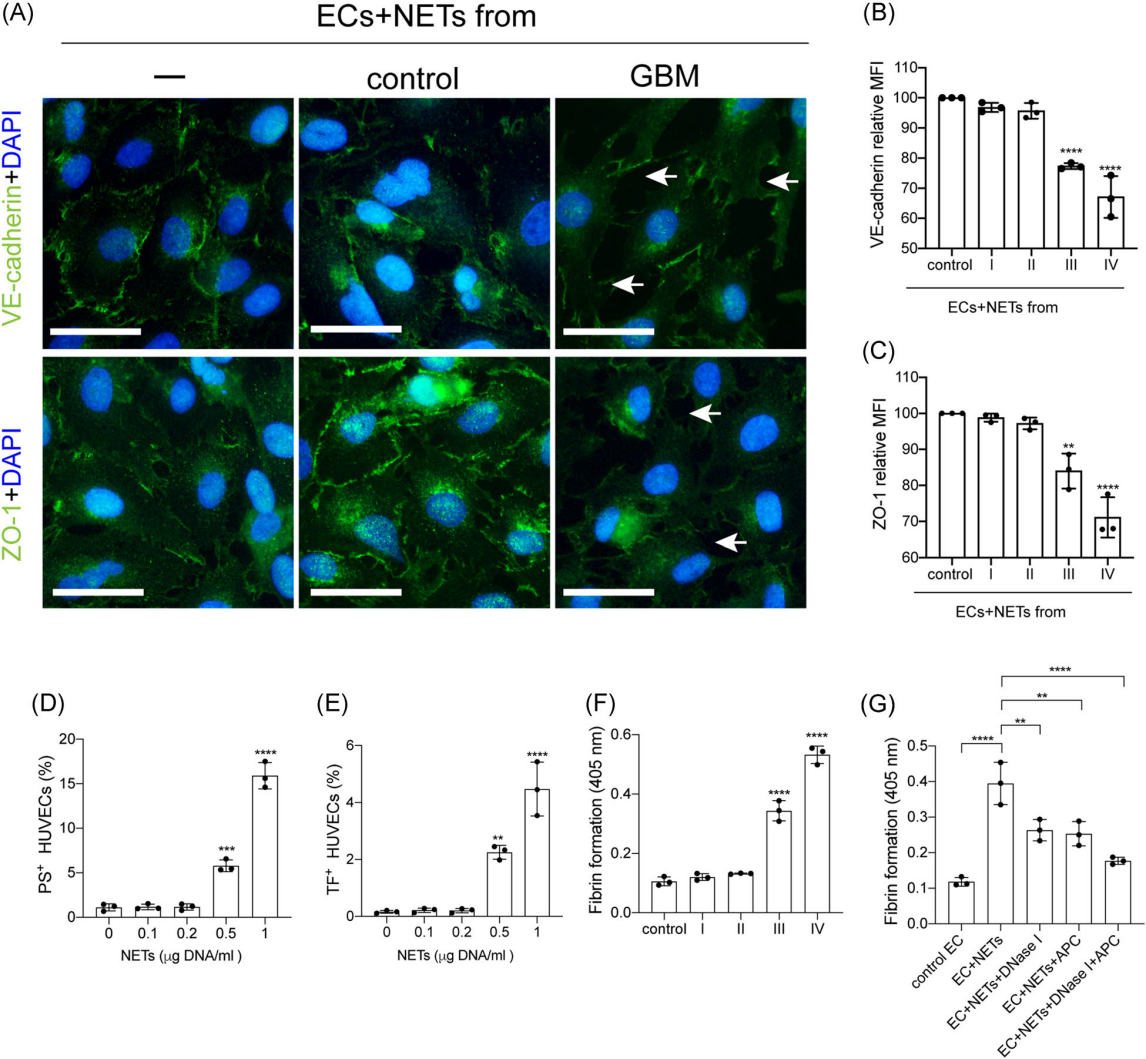
NETs convert endothelial cells (ECs) to a procoagulant phenotype. (A–C) ECs were incubated with NETs isolated from glioma patients and healthy subjects. The expression of VE‐cadherin and ZO‐1 on ECs were decreased after treatment with NETs from stage III/IV glioma patients. Expression is indicated as mean fluorescence intensity (MFI). The expression of PS (D) and tissue factor (TF) (E) on ECs were detected by flow cytometry in different concentrations of NETs. The expression of TF and PS on ECs were elevated with the increase of NET concentrations. (F) Fibrin formation of EC‐incubated NETs from glioma patients were measured. (G) ECs were incubated with isolated NETs in the presence of DNase I and APC. Fibrin formation of ECs was attenuated by DNase I and APC. The scale bar in (A) is 40 μm. The results are expressed as the mean ± SD. *p < .05, **p < .01, ***p < .001, and ****p < .0001. APC, activated protein C; NET, neutrophil extracellular trap; PLT, platelet; PS, phosphatidylserine

**Figure 5:**
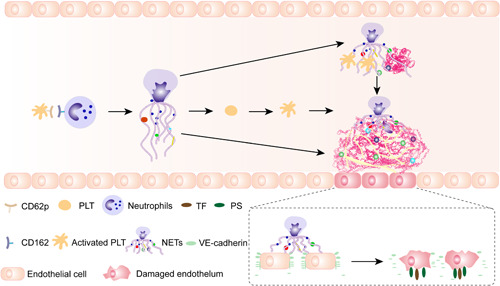
A model of the process of neutrophils extracellular trap formation in glioma patients and its interaction with endothelial cells and platelets. NET, neutrophil extracellular traps; TF, tissue factor; PS, phosphatidylserine

To investigate the interaction between NETs and ECs in glioma patients, ECs were incubated with NETs isolated from glioma patients and healthy subjects. The expression of VE‐cadherin and ZO‐1 on ECs decreased after treatment with NETs from stage III/IV glioma patients (Figure 4A–C). Tissue factor (TF) and PS expressions on ECs were detected by flow cytometry in different NET concentrations (Figures 4D and 4E). The expression of TF and PS on ECs increased with the increase of NET concentrations. We also detected fibrin formation of ECs incubated with NETs from glioma patients (Figure 4F). NETs from stage III/IV glioma patients converted ECs to a more procoagulant phenotype. Moreover, the PCA of ECs was attenuated by DNase I and APC (Figure 4G). Figure 5 shows a model of the process of neutrophils extracellular trap formation in glioma patients and its interaction with endothelial cells and platelets.

## 4 DISCUSSION

Four interesting findings were observed in the present study. First, GBM patients showed the highest levels of NET markers in plasma samples. Second, PLTs were critical initiators of NET generation in glioma patients. Third, NETs induce a hypercoagulable state in glioma patients. Finally, NETs convert ECs to a procoagulant phenotype.

Neutrophils have been identified as key players in innate immunity.^11^ In recent years, abundant clinical and basic studies confirmed that neutrophils participate in cancer pathophysiology which called tumor‐associated neutrophils (TAN).^12^, ^13^, ^14^ Moreover, neutrophil counts have previously been associated with the prognosis of glioma patients.^15^, ^16^, ^17^, ^18^ NETs have been recently reported in glioma tissues and their expression was correlated with glioma grades.^19^, ^20^ In our study, plasma levels of NETs were detected in glioma patients. NET markers in plasma from stage III/IV patients, especially GBM patients, were significantly higher than those from stage I/II patients and healthy subjects. IF images also confirmed the highest capacity of NET formation in neutrophils from GBM patients. Then we investigated the initiator of NET formation in glioma patients. Control neutrophils treated with PRP from GBM patients showed an increased tendency to expel NETs than those from the other groups. IF images also suggested potential PLT‐NET interactions in GBM samples. PLTs have been shown to play a critical role in NET generation in some cases.^36^, ^37^, ^38^, ^39^ HMGB1 originated from PLTs‐activated neutrophils to release extracellular traps in colon cancer.^26^ Our results suggested abnormal levels of PLT‐neutrophil aggregates in stage III/IV glioma patients, indicating the potential role of PLTs in NETs generation. To confirm our hypothesis, control neutrophils were incubated with PLTs from glioma patients. GBM PLTs activated neutrophils, resulting in higher levels of NETs. Anti‐p‐selectin and PSGL‐1 antibodies were used to inhibit PLTs‐neutrophils interaction^40^ and generation of NETs was significantly decreased.

It is well recognized that cancer patients are prone to develop venous thrombosis.^1^, ^2^, ^3^, ^4^, ^5^ VTE is a common vascular complication in glioma patients, occurring in 20%–30% of patients with HGG.^6^, ^7^, ^8^, ^9^, ^10^ However, the mechanism of VTE development in glioma patients is yet to be elucidated. NETs have been investigated in various thrombotic diseases such as stroke and myocardial infarction, as well as cancer‐associated thrombosis.^41^, ^42^, ^43^ In our study, we identified the hypercoagulable state in stage III/IV glioma patients, especially GBM patients, consistent with previous studies.^6^, ^7^, ^8^ Moreover, we found that the TAT complex was positively correlated with NET markers in the plasma from stage III/IV glioma patients, indicating the potential PCA of NETs in glioma patients. The results from fibrin formation showed that NETs can induce thrombogenicity in glioma patients. PLT activation was essential in thrombosis. Activated PLTs PS exposure contributes to hypercoagulability in colon cancers.^26^ Therefore, we investigate PCA on PLTs by NETs from glioma patients. Flow cytometry showed that the expression of PS on PLTs was significantly elevated when incubated with NETs from HGG patients. We also observed that NET‐treated PLTs were converted to a procoagulant phenotype, which could be responsible for the hypercoagulable state in glioma.

Endothelial injury is a critical pathogenic mechanism for thrombosis. We investigate the interaction between the abnormal generation of NETs and ECs. Our results showed that NETs from glioma patients destroyed the endothelial barrier and decreased the expression of VE‐cadherin and ZO‐1. Moreover, we found that NET‐treated ECs were converted to a procoagulant phenotype with the expression of TF and PS. NETs have been reported to exert a cytotoxic effect on EC death through histones and this effect could be reversed by APC.^26^, ^31^ In our study, by performing DNase I and APC to digest NETs, PCA of endothelial injury was significantly decreased. To the best of our knowledge, this is the first study proposing the potential role of endothelial activation and its integration with NETs in thrombogenicity in glioma patients. Our results may provide a new direction for the prevention and treatment of VTE in glioma patients.

However, there are some limitations in our study. First, our data was based on a small size sample from our hospital. Therefore, further studies with larger sample sizes should be conducted to ratify our conclusions. Second, our studies were based on samples from clinical patients and preliminarily investigated the interaction between NETs and hypercoagulability in glioma patients. Thus, animal experiments should be considered in the future to further verify our findings.

In summary, our study showed that NETs participate in the hypercoagulable state in glioma patients. We also revealed the interaction between NETs and PLTs activation and endothelial injury. The present study could offer potential therapeutic targets for the prevention and treatment of glioma complications.

## AUTHOR CONTRIBUTIONS

Shihua Zhang, Mengfan Guo, and Yankun Cui designed the study, analyzed the data, made the figures, and wrote the paper. Qianzi Liu and Jingfeng Liu conducted the assays and provide the study materials or patients. All authors approval of final manuscript.

## CONFLICT OF INTERESTS

The authors declare that there are no conflict of interests.

## Supporting information

Supplementary information.

## Data Availability

All data included in this study are available upon request by contact with the corresponding author.
