# Supplementary material for: Neutrophil extracellular traps induce a hypercoagulable state in glioma
Source: Immun Inflamm Dis. 2021 Jul 19;9(4):1383–93. doi: 10.1002/iid3.488 (PMC8589396; doi:10.1002/iid3.488)

**Supplement Figure 1**

(A) The flow cytometry strategy of PS^+^platelets from whole blood. (B) The flow cytometry strategy of platelet-neutrophil aggregates.


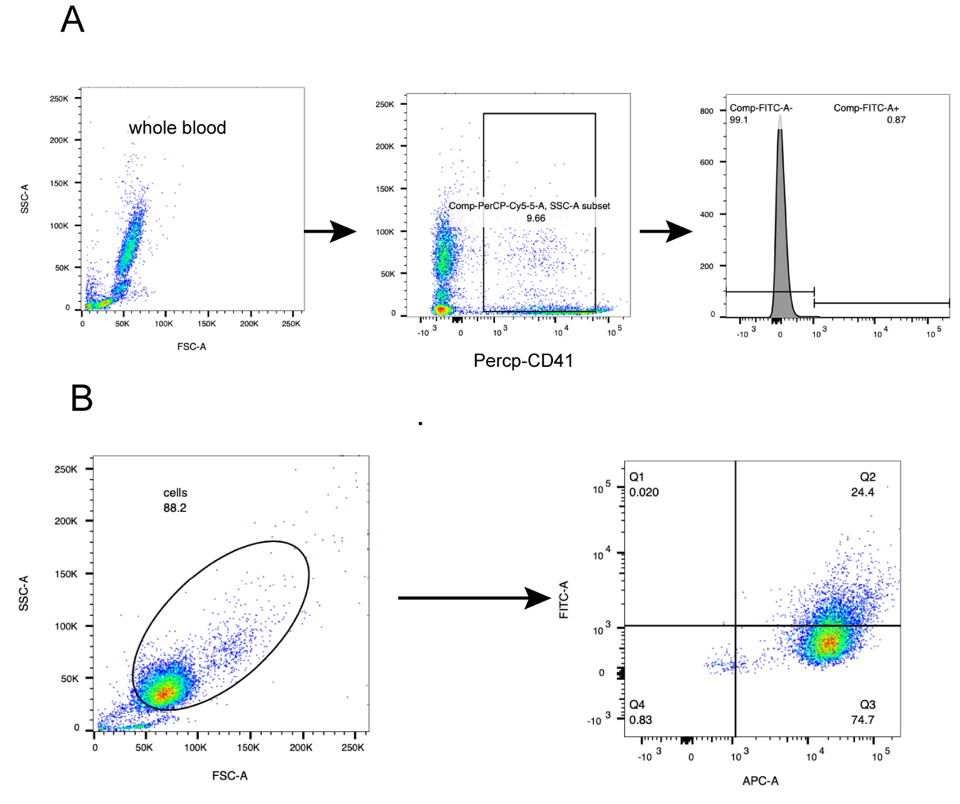


**Supplement Figure 2**

(A) The flow cytometry results of PS^+^platelet from each group. (B)The rate of PS^+^ platelet from each group. The results are expressed as the mean ± SD.*P<0.05, **P<0.01, ***P<0.001 and ****P<0.0001.


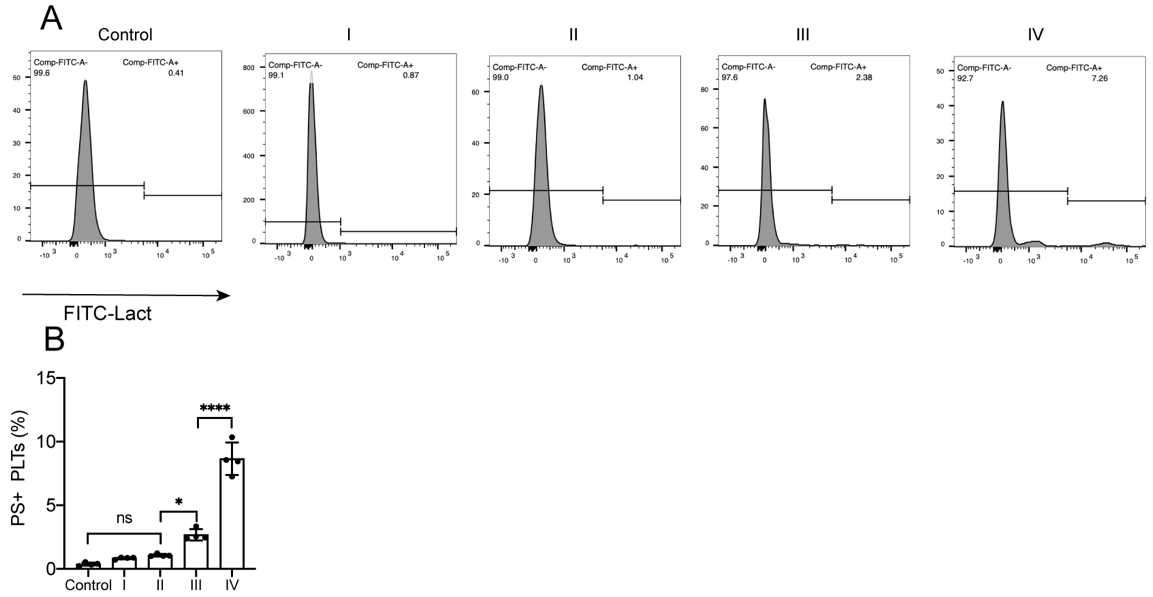

Supplement: Supplementary file 1 — Supplementary information. [file IID3-9-1383-s001.docx]
